# Exploring critical factors influencing physicians’ acceptance of mobile electronic medical records based on the dual-factor model: a validation in Taiwan

**DOI:** 10.1186/s12911-014-0125-3

**Published:** 2015-02-07

**Authors:** Chung-Feng Liu, Tain-Junn Cheng

**Affiliations:** Department of Information Management, Chia Nan University of Pharmacy and Science, No.60, Erh-Jen Rd., Sec.1, Jen-Te District, Tainan City, 71710 Taiwan; Department of Neurology, Occupational Medicine, Management in Medical Records and Information, Chi Mei Medical Center, No.901, Chung-Hwa Rd., Yong-Kang District, Tainan City, 710 Taiwan; Department of Occupational Safety/Institute of Industrial Safety and Disaster Prevention, College of Sustainable Environment, Chia Nan University of Pharmacy and Science, No.60, Erh-Jen Rd., Sec.1, Jen-Te District, Tainan City, 71710 Taiwan; Department of Occupational and Environmental Medicine, National Cheng Kung University Hospital, No.138, Sheng Li Road, Tainan City, 704 Taiwan

**Keywords:** Dual-factor model, Perceived threat, Perceived mobility, Mobile electronic medical records, Physicians

## Abstract

**Background:**

With respect to information management, most of the previous studies on the acceptance of healthcare information technologies were analyzed from “positive” perspectives. However, such acceptance is always influenced by both positive and negative factors and it is necessary to validate both in order to get a complete understanding. This study aims to explore physicians’ acceptance of mobile electronic medical records based on the dual-factor model, which is comprised of inhibitors and enablers, to explain an individual’s technology usage. Following an earlier healthcare study in the USA, the researchers conducted a similar survey for an Eastern country (Taiwan) to validate whether perceived threat to professional autonomy acts as a critical inhibitor. In addition, perceived mobility, which is regarded as a critical feature of mobile services, was also evaluated as a common antecedent variable in the model.

**Methods:**

Physicians from three branch hospitals of a medical group were invited to participate and complete questionnaires. Partial least squares, a structural equation modeling technique, was used to evaluate the proposed model for explanatory power and hypotheses testing.

**Results:**

158 valid questionnaires were collected, yielding a response rate of 33.40%. As expected, the inhibitor of perceived threat has a significant impact on the physicians’ perceptions of usefulness as well as their intention to use. The enablers of perceived ease of use and perceived usefulness were also significant. In addition, as expected, perceived mobility was confirmed to have a significant impact on perceived ease of use, perceived usefulness and perceived threat.

**Conclusions:**

It was confirmed that the dual-factor model is a comprehensive method for exploring the acceptance of healthcare information technologies, both in Western and Eastern countries. Furthermore, perceived mobility was proven to be an effective antecedent variable in the model. The researchers believe that the results of this study will contribute to the research on the acceptance of healthcare information technologies, particularly with regards to mobile electronic medical records, based on the dual-factor viewpoints of academia and practice.

**Electronic supplementary material:**

The online version of this article (doi:10.1186/s12911-014-0125-3) contains supplementary material, which is available to authorized users.

## Background

It is a common concern for industry, government, and academia to improve the quality of medical services, increase the safety of patients, and reduce medical costs through the use of information technologies, which also enhance competitiveness. Since the national health insurance system was launched in Taiwan in 1995, medical institutions have become more active in introducing a variety of technologies in order to get fee payments from the Bureau of National Health Insurance quickly and correctly. These technologies are related to healthcare information, such as computerized physician order entry systems (CPOE), medication administration systems, and clinical support systems. As these information technologies have evolved, innovative applications in the healthcare industry have become a global success. In recent years, the government of Taiwan has made great efforts to promote the development of electronic medical records (EMR), invested huge sums in subsidies, and instigated another great leap forward in healthcare information technology. For instance, medical coaching institutions have introduced ISO27001 information security certification and an electronic signature system, and the National Exchange Center of Electronic Medical Records has been established.

However, although medical institutions have introduced many new types of technology and systems and have spent large sums of money in the process of bringing about different levels of change to healthcare practice, questions and doubts remain as to whether they have yielded the expected benefits. For example, a study by Lærum and colleagues [1] discovered that physicians only used a small percentage of the functions constituting an EMR system. A discussion on the introduction of healthcare information technology (HIT) showed that the anxiety of healthcare professionals was always an important influencing factor [2]. During the introduction of innovation technologies, healthcare professionals need to not only change their working customs but also learn to adapt, which has an impact on their work. As a result, the resistance of physicians to new technologies has long been considered a common problem during the introduction of healthcare information systems in medical institutions [3,4].

With respect to information management, most of the previous studies on the acceptance or adoption of innovation technologies were carried out from “positive” perspectives, such as perceived usefulness and perceived ease of use, with regards to the Technology Acceptance Model (TAM) [5]. Only a few studies have adopted systematic methods (such as model validation) to discuss the negativity of users toward innovation technologies, such as perceived threats [6], innovation resistance [7], and technophobia [8]. The situation is the same in the healthcare field [9-14]. As the application of information technologies is always influenced by both positive and negative factors, it is necessary to validate both to gain a complete understanding. This is the basic concept of the dual-factor model, considering both positive and negative factors. Based on this concept, Walter and Lopez [15] introduced the new negative factor of “perceived threat to professional autonomy” (“perceived threat” for short) based on the TAM in order to discuss US physicians’ acceptance of EMR and Clinical Decision Support (CDS). They discovered that “perceived threat” influenced physicians’ perceptions of the usefulness of information technologies as well as their intention to use these technologies. This study believes that it is worthwhile to validate the model proposed by Walter and Lopez’s [15] research model for Eastern countries and confirm the general explanatory power of the model.

Information and communication technologies are well developed in Taiwan [16], and the development of EMR in hospitals is also comparatively mature [17]. Consequently, it is proper for this study to choose Taiwanese physicians as the research subjects to validate the research model proposed in Walter and Lopez’s study [15]. Since Taiwan started to promote its national health insurance system in 1995, traditional (desktop and wired) EMR has been commonly used in hospitals [17]. In addition, mobile healthcare (also known as m-Health and m-Healthcare) is considered to have significant benefits and is in the stage of initial development [18]. Nevertheless, presenting ubiquitous services to healthcare professionals is not easy. A key challenge is progressing m-Health approaches from pilot projects to wider implementions whilst properly engaging healthcare professionals in the process [18]. Developers of these projects need to expend substantial effort and resources to ensure mobile service support. Thus, understanding the factors that influence healthcare professionals’ usage of mobile services is important to the development of mobile electronic medical records (MEMR). Therefore, two research questions are presented in this study: 1) Is the dual-factor model proposed by Walter and Lopez [15] applicable for evaluating physicians’ acceptance of MEMR in Eastern countries? 2) Could the feature of “perceived mobility” become a valuable antecedent variable for each of the inhibitors and enablers in Walter and Lopez’s model [15]?

### Mobile electronic medical records

Various definitions for computer-based patient records have been advanced e.g., [19,20]. A consentaneous definition of EMR provided in the US National Alliance for Health Information Technology Report to the Office of the National Coordinator for Health Information Technology [21] states that “EMR is an electronic record of the health-related information on an individual that can be created, gathered, managed, and consulted by authorized clinicians and staff within one healthcare organization.” In Taiwan, the core law source for electronic medical records is the *Medical Care Act*, which was promulgated on November 24, 1986. According to updated Article 69 of the *Medical Care Act*, “medical records that are generated and stored in the form of electronic files are exempted from generating paper versions, and their qualifications, generating methods, content, and other matters to follow shall be determined by the competent central authority”. In the past ten years, in order to speed up the development of electronic medical records, the Department of Health has undertaken several measures, such as revising the related laws and regulations, generating standards, providing technological support, strengthening information security, and providing subsidies. It has also ensured the implementation of paperless medical records during hospital accreditation and healthcare inspections so as to encourage a hospital’s initial intention to use electronic medical records.

Due to the flourishing of wireless communication networks and the rapid evolution of handheld electronic devices, the mobility and “wirelessness” of electronic medical records have become more feasible. Ying believed that the Mobile Physician Order Entry should focus on certain desired features, such as high-yield orders, a simple interface, and mobility [22]. A Wireless Health Outcomes Monitoring System (WHOMS) was developed and tested with cancer patients using mobile phones and the results suggested that such a mobile system has the potential to detect patient-suffering earlier and enable the start of well-timed intervention [23]. Wu and colleagues pointed out that physicians who used PDA to carry out Computerized Physician Order Entry (CPOE) felt it was necessary to enhance the response speed of the system, simplify operations, and improve the display, and so on [24]. Hsieh and colleagues discussed the idea that the adoption of Mobile Electronic Medication Administration Records can reduce human error and enhance medication safety [25].

The aforementioned studies were more concerned with the application, advantages, and disadvantages of the mobile electronic medical record, and seldom gave a clear definition of a mobile electronic medical record. Therefore, referring to the definition by Hsu and colleagues [26], this study defines a “mobile electronic medical record” as “an EMR that can be accessed and managed through mobile computers to help physicians deliver health care anytime and anywhere”. During a physician’s clinical practice, a mobile electronic medical record means the use of a mobile device (such as a tablet computer, laptop, mobile phone or PDA) that can be taken on rounds, used during inspections and consultations, used for enquiries about physician orders and prescriptions, and for the performance of other routine physician duties.

### Prior research and hypotheses

#### The dual-factor model that influences the intention to use technology

A rather high proportion of the studies on information systems (IS) discussed influences on the adoption, acceptance, and usage of such systems e.g., [5,27-31]. Many studies analyzed the belief in the system adoption, satisfaction with the system, and other factors that would promote the success of the system, lead to positive attitude, and encourage usage. However, there are comparatively fewer studies on the hindrances or inhibitions of system usage [32]. Cenfetelli believed that negative factors (inhibitors) and positive factors (enablers) were the external beliefs of users toward system features, which would influence users’ decisions when adopting or refusing the system [32]. Therefore, it is necessary to consider these factors.

Walter and Lopez [15] posited that the perceived threat to professional autonomy is a salient outcome belief affecting physician acceptance of an HIT. Including this negative factor in the TAM [5], as well as the core positive constructs of perceived ease of use and perceived usefulness, they proposed and validated a comprehensive model to explain physicians’ acceptance of HITs in the USA. Their model suggests that perceived usefulness, perceived ease of use and the perceived threat of HIT are major determinants of behavioral intention. Furthermore, perceived usefulness is influenced by perceived threat and perceived ease of use.

The following sections will discuss and comment on the positive and negative influencing factors in Walter and Lopez’s [15] model. In addition this study introduces and discusses “perceived mobility” as a potential antecedent variable in the model.

### Perceived usefulness and perceived ease of use

The Technology Acceptance Model (TAM) is one of the best known theories in the modern information system research field and is frequently cited [33]. It was developed by Davis and colleagues in 1989 [5], referring to the Theory of Reasoned Action [34] and the Theory of Planned Behavior [35], and was used to explain the relationship between technology and user behavior [5]. The TAM inherits the basic idea of the Theory of Reasoned Action and states that internal beliefs will influence “attitude”, which will further influence the intention for usage. The intention for usage has a significant and positive impact on the actual use of the system.

Szajna [36] removed the variable of user’s “attitude” from the original TAM and revised the model to conclude that the intention to use is strong enough to influence technology acceptance. The study divided the TAM into two models, before and after the actual operation. The greatest contribution of the TAM lies in the introduction of two perceived beliefs, (perceived ease of use and perceived usefulness) that influence the users’ technology acceptance. These two constructs are the most widely used positive factors.

Later, Venkatesh and Davis [27] proposed TAM 2, and Venkatesh and Bala [29] proposed TAM 3. Both of these models are still based on the two core beliefs of perceived ease of use and perceived usefulness, but the difference is that they add broader external variables. The applicability of TAM to the HIT field has been widely validated [33], including the healthcare field [37]. This study therefore proposes the basic hypotheses of the TAM:

- H1: Physicians’ perceived usefulness positively influences their intention to use MEMRs.
- H2: Physicians’ perceived ease of use positively influences their intention to use MEMRs.
- H3: Physicians’ perceived ease of use positively influences their perceived usefulness of MEMRs.

### Perceived threat

The acceptance of a new technology is often mired by a reluctance to forsake the commitment to a previous work configuration and the perception of threat to continued job security (e.g. loss of power) [6].

Harvey [38] posited that physicians’ resistance to the introduction of information technology is common, and that the primary issue is the collection of information can be threatening individually, as the potential for peer review or performance review by managers is obvious.

Physicians are particularly sensitive to changes in their working environment that will threaten their working autonomy [39,40]. They feel uncomfortable knowing that other people can ascertain information about their care of their patients [41]. The so-called ‘other people’ also include the computer system. Therefore, physicians may be reluctant to use a computer system [41] or have a tendency to deny the usefulness of the system.

Walter and Lopez [15] defined professional autonomy as professionals’ having control over the conditions, processes, procedures, or content of their work according to their own collective and, ultimately, individual judgment in the application of their profession's body of knowledge and expertise. Thus the perceived threat of IT to professional autonomy (also abbreviated as perceived threat in this study) can be defined as the extent to which professionals perceive that IT systems will threaten their professional autonomy. In health care, physicians may perceive that MEMRs transgress their professional autonomy and are not useful due to the belief that they can conduct the best decision-making for patient-care without MEMR assistance. As a result, they may express a low intention to use MEMRs.

Walter and Lopez’s study on US physicians’ usage of HITs [15] confirmed that “perceived threat” had a negative influence on perceived usefulness, as well as on physicians’ intention to use the system. A similar study exploring consumers’ health behavior intention also validated the causal relationship between perceived threat and usefulness [42]. Therefore, the following hypotheses are proposed:

- H4: Physicians’ perceived threat negatively influences their perceived usefulness of MEMRs.
- H5: Physicians’ perceived threat negatively influences their intention to use MEMRs.

### Perceived mobility as an effective universal antecedent

Mobility enables users to receive and transmit information anytime and anywhere. Computer-supported collaborative work and human/computer interactions have provided an insight into the characteristics, requirements, and implications of mobile technology use. This mobility (also known as ubiquity) means that, with the help of mobile terminals and networks, users can access mobile services, such as mobile banking, anytime and anywhere [43]. Compared with traditional e-commerce, mobile computing provides access to information, communication, and services that are independent of time and place [44]. Thus, mobility, in the study by Mallat and colleagues [44], in the e-ticketing context is used to express the benefits of time and place, service access, and usage. Mobility is perceived to be the most significant feature of a mobile service.

Huang and colleagues [45] explored user behavior in mobile learning and found that perceived mobility has a positive impact on perceived usefulness. Such a causal relationship has also been validated in other contexts, such as consumers’ acceptance of mobile payment services [46], users’ employment of mobile map services [47] and players’ acceptance of mobile social network games [48]. In healthcare, mobility can express the same benefits for healthcare professionals as well. It frees them from spatial and temporal limitations and enables them to conduct ubiquitous healthcare, especially at the point of care. Of course, physicians may also realize that MEMRs are useful tools for care purposes. Thus, the following hypothesis is proposed:

- H6: Physicians’ perceived mobility positively influences their perceived usefulness of MEMRs.

Due to the technical limitations of mobile devices, ease of use becomes an imminent acceptance driver for mobile applications. Some of the limitations include small screen size, low battery life and reduced input/output capabilities. This is especially true for mobile services, which compete with established solutions and thus need to provide benefits when it comes to ease of use [46]. However, mobile services enable users to access information and people anytime and anywhere. Consequently, this feature enhances the ease of use of the service. Two recent studies regarding users’ acceptance of mobile services have provided preliminary evidence, which have confirmed the existence of the relationship between perceived mobility and perceived ease of use [49,50]. In healthcare, mobility can be regarded as the main advantage of MEMRs compared to traditional EMRs. If physicians perceive that MEMRs have higher mobility, it means that they can access MEMRs easier at any time and from anywhere, especially at the point of care. Thus, the following hypothesis is proposed:

- H7: Physicians’ perceived mobility positively influences their perceived ease of use of MEMRs.

Steinhubl [51] states that the level of exuberance for m-Health is driven by the convergence of three powerful forces, one of which is the need for more precise and individualized medicine that enables physicians to have more control and time to complete their care work. In clinical practice, mobility of MEMRs enables the transmission of patient information to physicians without space or time limitations. When physicians have received or are inquiring about important information, they are able to conduct succeeding disposition and make decisions immediately, which will reduce the interference or threat to their control or the autonomy of their medical treatment. Although the academic field hasn’t yet studied the influences of perceived mobility on perceived threat, this study believes that both of the variables should have a proper relationship in medical practice. Therefore, the following hypothesis is proposed:

- H8: Physicians’ perceived mobility negatively influences their perceived threat of MEMRs.

## Methods

### Research framework

**Figure 1:**
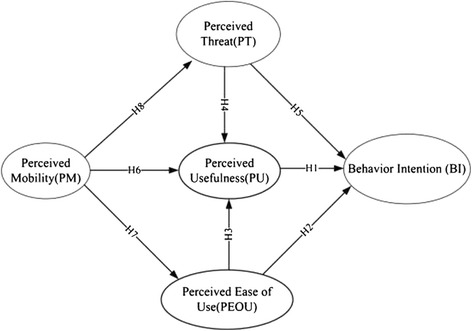
**Research framework.**

The framework of this study was constructed by mainly referring to related theoretical ideas, such as the dual-factor model and the Technology Acceptance Model, and was used to discuss factors that influence a physician’s usage of medical information technologies. The research framework is shown in Figure 1. The constructs and variables of this study come from the aforementioned literature. The operational definitions of the independent and dependent variables are shown in Table 1.

**Table 1:** **Operational definitions of variables**

**Variable**	**Operation definition**	**Reference**
Behavior Intention (BI)	The strength of a physician's intention to use MEMR.	Davis et al. 1989 [5]; Davis 1989 [52]
Perceived Usefulness (PU)	The extent to which a physician believes that using a MEMR would enhance their care performance.	Davis et al. 1989 [5]; Davis 1989 [52]
Perceived Ease of Use(PEOU)	The extent to which a physician believes that using MEMR would be free of effort.	Davis et al. 1989 [5] Davis 1989 [52]
Perceived Threat(PT)	The extent to which a physician believes that using the MEMR would decrease their control over the conditions, processes, procedures, or content of their care work.	Walter & Lopez 2008 [15]
Perceived Mobility(PM)	The extent to which a physician can access MEMR at any time and from anywhere.	Zhou 2012 [43]

### The design of the questionnaire

The design of the questionnaire for this study was based on related theories and questionnaires developed and validated by previous scholars. Therefore, it is believed that the questionnaire designed for this study has high reliability and validity. Firstly, the literature was reviewed to collect measuring tools that had been rigorously validated, and these were then used as the foundation to develop the questionnaire for this study. The questionnaire was then translated into Chinese, and the sentences modified to obtain the first draft of the questionnaire. After that, three experts (a professor with a PhD in the medical information field, a professor with a PhD in the medical management field, and a clinical physician with a PhD) were asked to verify the draft and evaluate whether the sentences and their meanings were properly expressed (pretesting), and whether they could be merged for simplification. Finally, three potential users (physicians) were asked to complete the revised questionnaire as a pilot test, before the questionnaire for this study was finalized (Additional file 1).

In this study, the items for measuring perceived threat were adapted from Walter and Lopez’s instrument [15], the items for measuring perceived mobility were adapted from Lee’s instrument [53], while the instruments for perceived ease of use, perceived usefulness, and behavioral intention were adapted from previous empirically validated studies [9,5,52]. The variables and corresponding measurement items of this study, as well as reference resources, are shown in Table 2.

**Table 2:** **Measurement items of the variables and reference resources**

**Variable**	**Measurement Items**	**Reference**
BI	1. If the hospital decides to develop MEMR in the future, I shall frequently use it.	Hu et al. 1999 [9]
	2. If the hospital decides to develop MEMR in the future, I will use it to assist my healthcare work.	
	3. I think I will recommend other physicians (from this hospital or not) to use MEMR.	
	4. If the hospital decides to develop MEMR in the future, it will become one of my favorite assistance tools for my work.	
PU	1. Using MEMR will speed up my work (e.g. going on rounds and consulting medical records).	Davis et al. 1989 [5]; Davis 1989 [52]
	2. Using MEMR will improve my work quality (such as enhancing the immediacy of prescribing physician orders).	
	3. Using MEMR will make it easier to conduct my work.	
	4. Using MEMR will improve my working performance.	
	5. Using MEMR will help me to control my work better.	
PEOU	1. It is easy to understand the operations of MEMR.]	Davis et al. 1989 [5]; Davis 1989 [52]
	2. It is easy to use MEMR to finish my work.	
	3. On the whole, MEME is easy to use.	
PT	1. Using MEMR may decrease my control over clinical decisions.	Walter & Lopez 2008 [15]
	2. Using MEMR may decrease my professional discretion over patient care decisions.	
	3. Using MEMR can decrease my control over each step of the patient care process.	
	4. Using MEMR may increase the monitoring of my diagnostic and therapeutic decisions by non-providers.	
	5. Using MEMR may decrease my control over the allocation of scarce resources.	
	6. I would find MEMR advantageous for the medical profession as a whole.	
PM	1. I can access the MEMR at any time for the necessary information or service for my patient care	Lee 2005 [53]
	2. I can access the MEMR anywhere for the necessaryzinformation or service for my patient care	
	3. I can use the MEMR “anywhere,” and “anytime” at the point of patient care.	

### Study subjects and ethical considerations

The study subjects are the entire roster of physicians from three branch hospitals of a medical center (one is a hospital at medical center level, one is a hospital at regional level, and one is a hospital at district level), including resident physicians, attending physicians, and concierge physicians. In Taiwan, the hospital accreditation system, as implemented in 1978 by Taiwan’s Ministry of Education and Department of Health (DOH), issues a level of accreditation, determined by the size, capabilities, and performance quality of a hospital. Based on these accreditation rules, the three main levels are categorized as medical center, regional hospital and district hospital. Thus, the study subjects chosen are fully representative of the medical system in Taiwan.

In order to protect the rights and privacy of the participants, appropriate ethical approval for this study was obtained from the Institutional Review Board of the hospital (Chi Mei Medical Center) before the questionnaires were officially distributed.

## Results

### Descriptive statistics

A total of 158 valid questionnaires were collected from the three hospitals. As the total number of physicians across the three hospitals is 473, the recovery rate of valid questionnaires was 33.40%. Among these questionnaires, 71.52% (113 copies) were from the medical center, with male respondents accounting for the majority (81.65%, 129 copies); the proportion from the Department of Internal Medicine was the highest (37.97%, 60 copies) (see Table 3). Table 4 shows that the respondents, with respect to MEMRs, gave extremely high approval for the perceived ease of use, usefulness, compatibility, and relevance to work (mean > 4). The respondents expressed a comparatively low level of perceived threat regarding MEMRs (2 < mean < 3).

**Table 3:** **Descriptive statistics of the respondents**

**Branch hospital**	**Copies of questionnaires returned**	**Return rate**
The Medical Center	113	71.52%
The Regional Hospital	27	17.09%
The District Hospital	18	11.39%
**Gender**	**Copies of questionnaires returned**	**Return rate**
Male	129	81.65%
Female	21	13.29%
N/A	8	5.06%
**Department**	**Copies of questionnaires returned**	**Return rate**
Internal Medicine	60	37.97%
Surgery	36	22.78%
Gynecology and Pediatrics	19	12.03%
Emergency and Critical Care Medicine	15	9.49%
Others	28	17.72%

**Table 4:** **Descriptive statistics of the criteria for determining the quality of the responses**

**Construct**	**Mean**	**SD**	**CR**	**Cronbach’s alpha**	**AVE**	**Item**	**Factor loading**
BI	4.15	0.65	0.94	0.92	0.81	BI1	0.91
						BI2	0.92
						BI3	0.86
						BI4	0.91
PEOU	4.05	0.73	0.96	0.93	0.88	PEOU1	0.93
						PEOU2	0.94
						PEOU3	0.94
PT	2.11	0.76	0.97	0.96	0.82	PT1	0.92
						PT2	0.91
						PT3	0.91
						PT4	0.92
						PT5	0.87
						PT6	0.89
PU	4.37	0.67	0.97	0.96	0.86	PU1	0.91
						PU2	0.91
						PU3	0.95
						PU4	0.95
						PU5	0.91
PM	4.24	0.72	0.93	0.89	0.82	PM1	0.94
						PM2	0.91
						PM3	0.88

### Reliability and validity

Cronbach’s α for each of the constructs was greater than 0.9, exceeding the suggested cut-off value of 0.7, and the composite reliability (CR) of all constructs exceeded the suggested cut-off value of 0.6. These results all indicated that the measurements satisfied the reliability criteria [54]. Fornell and Larcker [55] suggested using the average variance extracted (AVE) as a measure of convergent validity. Table 4 demonstrates that the AVEs ranged between 0.81 and 0.88, exceeding the cut-off value of 0.5 [55], suggesting satisfactory convergent validity. Additionally, Table 5 shows that none of the construct intercorrelations exceeded the square root of the AVE of the constructs, establishing discriminant validity [55]. Overall, all of the constructs in this study exhibited sufficient convergent and discriminant validity.

**Table 5:** **Correlation matrix**

	**BI**	**PEOU**	**PT**	**PU**	**PM**
**BI**	**0.90**				
**PEOU**	0.72	**0.94**			
**PT**	−0.47	−0.37	**0.91**		
**PU**	0.71	0.73	−0.43	**0.92**	
**PM**	0.43	0.51	−0.22	0.49	**0.91**

Note: The bold numbers on the leading diagonal show the square root of the variance shared by the constructs and their measures.

The partial least squares (PLS) technique was used in this study to evaluate the measurement and structural models [56]. The principal component analysis of the PLS was performed to ensure the unidimensionality of the three constructs PU, PEOU, and PT. The factor loadings of all of these items were equal to or greater than 0.86, which exceeded the cut-off of 0.7 suggested by Fornell and Larcker [55]. These were significantly associated with only one latent variable, indicating conformance to unidimensionality [57].

According to the PLS path modelling structure, the measurement model, structural model and overall model need to be validated with three different fit indices, namely the communality index, the redundancy index and the Goodness of Fit (GoF) index [56]. GoF is employed to judge the overall fit of the PLS model, which is computed as the geometric mean of the average communality and the average R square. The redundancy index represents the amount of variance in an endogenous construct explained by its independent latent variables. High redundancy means a high ability to predict. A good value for the AVE index (equal to the communality index in the PLS analysis) is at least 0.50, which means that 50% or more of the variance is accounted for. GoF is normed between 0 and 1, where a higher value represents better path model estimations. For this model, the average redundancy value is 0.26, the average communality value is 0.84, and the GoF value is 0.56, which exceeds the cut-off value in comparison with the baseline value as GoFsmall =0.1, GoFmedium =0.25, GoFlarge =0.36 [58]. These indices indicate that this model has substantial predictive power.

### Hypotheses testing

**Figure 2:**
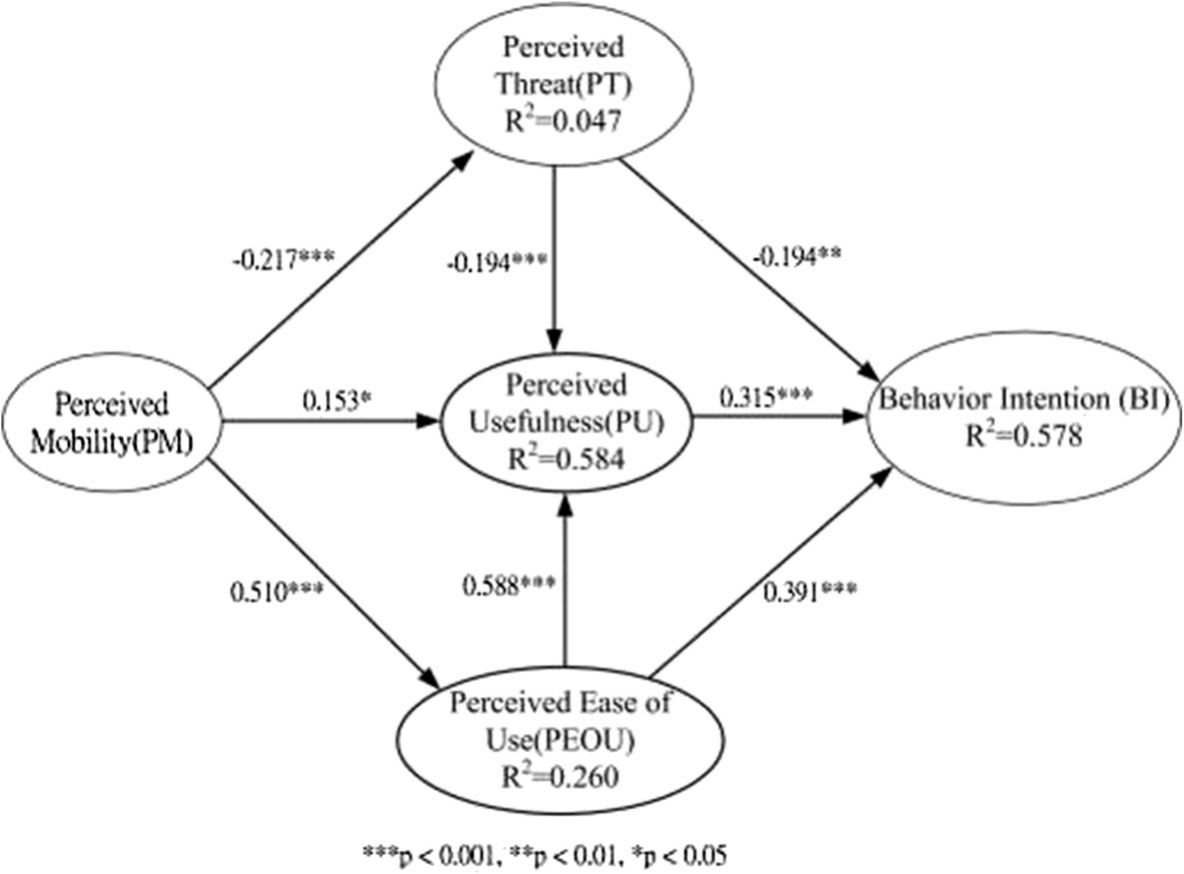
**PLS path analysis results.**

The statistical significance of the parameters in the structural model was tested using the bootstrapping resampling procedure of the PLS method. SmartPLS® 2.0 M3 software was used [59]. With a significance of 0.05 or better, the results revealed that the physicians’ perceptions of PU and PEOU were positively associated with their behavioral intentions to use MEMR, while PT was negatively associated. These three factors jointly explained about 57.8% of the variance. PEOU and PT associated with the common antecedent of PM explained approximately 58.4% of the variance in PU. Surprisingly, PM was confirmed to be an effective antecedent that solely influenced PT and PEOU with a lighter explanatory power of 4.70% and a stronger explanatory power of 26.0%, respectively. These results support all the hypotheses proposed. Figure 2 presents the standardized path coefficients of the causal path.

## Discussions and suggestions

### Discussions

This study has discovered that physicians, generally, have a high intention to use MEMRs (mean = 4.15), which indicates that, in Taiwan, the development of m-Health has been widely accepted by healthcare professionals. Another study targeting nursing staff also demonstrated the same result [26]. In Taiwan, due to the implementation of the national health insurance system, medical informatics has been well developed, and it is common for healthcare professionals to use information technologies to assist their work. In addition, the information and communication industry of Taiwan is quite advanced. All kinds of mobile facilities are available to and popular with the public. Therefore, physicians have a positive attitude toward the adoption of innovative technologies, such as MEMRs.

This study has verified the influence of perceived threat. That is, with respect to physicians’ acceptance toward innovation technologies, apart from the positive factors of usefulness and ease of use, the threat perceived by physicians should not be neglected. This study result and the study result of Walter and Lopez [15] have provided strong support to demonstrate that this phenomenon is no different between Western and Eastern countries. The study result shows that perceived threat has a significant influence on perceived usefulness. This indicates that physicians feel that MEMRs will threaten their working autonomy because MEMRs enable other people to acquire more information about the treatment of their patients. As a consequence, physicians may feel uncomfortable with this aspect and will then tend to reject the usefulness of MEMRs, leading to a decrease in their intention to use MEMRs.

With respect to positive factors, usefulness and ease of use have also been verified to have a direct influence on physicians’ intention to use MEMR, which conforms to the common experience generated from previous studies regarding individuals’ acceptance of new technologies.

To summarize, physicians’ intention to use MEMRs is still significantly and directly related to perceived ease of use and perceived usefulness. However, perceived threat, neglected by previous studies, has been proven by this study to have a negative influence on physicians’ adoption of MEMRs.

Another purpose of this study was to discuss whether perceived mobility is the antecedent variable of all the positive and negative factors. The study result demonstrates that perceived mobility does have a significant influence on perceived usefulness, perceived ease of use, and perceived threat. However, it is worth noting that perceived mobility only has a slight influence on perceived threat. This implies that other obscure factors, such as patient autonomy [60,61], may be closely associated to a physician’s perceived threat of professional autonomy. In clinical practice, a MEMR is able to transmit all kinds of useful and relevant information, such as data reports for examinations and tests, without space or time limitations. With this on-demand information, physicians are able to consult the necessary information for timely decision-making. In this way, physicians will suffer less mental anxiety while still feeling they have sufficient autonomy and control over their medical practice, and the threat they may feel towards MEMRs will be reduced. However, the feature of mobility is not a primary concern for increasing physicians’ professional autonomy.

### Suggestions

First, this study suggests that, when developing MEMRs, it is better not to over-emphasize the nature of the technology’s innovation and automation, such as providing too many tips or guidelines, as it will make physicians feel that their expertise or autonomy to make decisions regarding diagnosis is interfered with or challenged. As a result, it is suggested that, when developing MEMRs, it is necessary to safeguard the autonomy of medical practitioners. Otherwise, physicians may feel threatened and will then reject the system.

Secondly, MEMR developers should give sufficient consideration to the features (touch input, small screen, etc.) of mobile facilities, and pay primary attention to providing basic and necessary functions. They should not allocate too many functions to a limited screen window while also ensuring that physicians do not have to switch pages too frequently. The system should be made easy to operate, and the tools of the MEMR should be easily controllable just by sliding so as to avoid incorrect character input.

Third, this study suggests that industry, government, and academia should fully communicate with physicians to ensure MEMRs give full support to their medical practice while not hindering control over their medical decision-making. Otherwise, physicians’ intention to use MEMRs will decrease.

Finally, regarding the mobility feature, this study suggests that, while developing MEMRs, industry, government, and academia should pay attention to the availability and readiness of the EMR infrastructure, including increasing the coverage, stability, and speed of wireless networks at places of work. Therefore, attention should be paid to the effective maintenance and availability of mobile facilities (such as updating operating systems to fix bugs) and the readiness (such as sufficient power). Thus, the mobility of MEMRs will be enhanced, which will increase physicians’ perceived usefulness and perceived ease of use, and reduce their perceived threat regarding MEMRs.

## Conclusion

### Contributions and implications

In recent years, the development of innovation technology for m-Health has been a focus of attention. Although many clinical testing systems or academic research plans have proposed a number of interesting application prototypes, the technology is still not commonly used in medical practice. Also, as the development and maintenance of the application systems of MEMRs are expensive (such as costs for tablet devices, wireless networks, developing technologies and tools, user training, etc.), it is important that the industry, government, and academia understand the inhibitors and enablers that will influence physicians’ intention to use MEMRs during the development of m-Health. This study has discussed this research issue using the dual-factor model and has obtained a high explanatory power. It is valuable for accumulating research experience of the dual-factor model in the healthcare field. One of the contributions of this study is confirmation that, both in Western and Eastern countries, the perceived threat to professional autonomy is an important factor with regards to perceived usefulness in the context of physicians’ acceptance of HITs and has a significant, negative impact on their intention to use HITs. This implies that excessive use of technology may adversely affect user perception, even for highly intelligent knowledge workers, such as physicians. In addition, this study has also confirmed that usefulness and ease of use are still the two critical factors that influence healthcare professionals’ intention to use HITs. This study, therefore, reminds the healthcare industry, government, and academia to maintain attention on these two factors. Another contribution of this study is to validate perceived mobility as an effective antecedent variable while modelling physicians’ acceptance of mobile technology. This study calls for continuous exploration into perceived mobility in health care and other fields while initiating a mobile service. To summarize, it is worth stressing that previous studies have seldom discussed the influence of perceived mobility on perceived ease of use, perceived usefulness and perceived threat. Thus, this study reveals both academic and practical values in the healthcare field.

### Future research direction

Based on this study, future researchers can integrate other meaningful negative factors, such as perceived risk [62], to enhance the explanatory power of the research model of this study. This study discovered that perceived mobility was an effective antecedent variable of the factors that influence the intention to use mobile services. However, the low explanatory power of perceived mobility on perceived threat highlights the fact that a comprehensive literature review is still necessary in order to identify additional critical factors for follow-up research. In addition to perceived mobility, other valuable antecedent variables may exist and deserve to be explored in the future. In addition, some studies have discovered that perceived mobility itself is directly related to the intention to use mobile services [45,63,46]. It is worthwhile testing this difference. Another urgent aspect to explore is nurses, the largest group of care workers, and their perceived threat relating to MEMRs, mainly when using mobile nurse stations. Beyond places of care, mobile technologies also present an opportunity to connect patients and health workers so as to improve the quality of care given at the point of care and reduce unnecessary referrals [64]. Therefore, patients’ intention to accept MEMRs is also worthy of study.

### Limitations

Although this study attempted to be rigorous during the implementation, research limits may still exist. Firstly, as the study subjects are physicians from three hospitals under the same medical system, the extrapolation validity of the study results may be insufficient. Also, as the questionnaires of this study were completed by means of self-reporting, each respondent may have a different understanding or perception of the meanings of the questions, which may cause common method bias (CMB) or common method variance (CMV), and further influence the study results.

## Additional file

Additional file 1:
**Survey questionnaire.**
