# Supplementary material for: Exploring critical factors influencing physicians’ acceptance of mobile electronic medical records based on the dual-factor model: a validation in Taiwan
Source: BMC Med Inform Decis Mak. 2015 Feb 7;15:4. doi: 10.1186/s12911-014-0125-3 (PMC4333263; doi:10.1186/s12911-014-0125-3)
Supplement: Additional file 1: — Survey questionnaire. [file 12911_2014_125_MOESM1_ESM.doc]

Exploring Critical Factors Influencing Physicians’ Acceptance of Mobile Electronic Medical Records (MEMR)

**Part I. Respondent’s basic information.**

1. Gender: □Male □Female
2. Age(yrs): □<26 □26-30 □31-35 □36-40 □41-45 □46-50 □51-55 □56-60 □>60
3. Which hospital do you serve? □A □B □C
4. Department: □Internal Medical □Surgical □Gynecological and Pediatric □Emergency/ICU □Others ____________
5. Total work seniority(yrs)？____

**Part II. Question items.**

(Please tick the appropriate places depending on your agreement level.

| **descriptions** | Extremely agree | Somewhat  agree | Neutral | Somewhat  disagree | extremely disagree |
| --- | --- | --- | --- | --- | --- |
| 1. If the hospital decides to develop MEMR in the future, I shall frequently use it. | **□** | **□** | **□** | **□** | **□** |
| 1. If the hospital decides to develop MEMR in the future, I will use it to assist my healthcare work. | **□** | **□** | **□** | **□** | **□** |
| 1. I think I will recommend other physicians (from this hospital or not) to use MEMR. | **□** | **□** | **□** | **□** | **□** |
| 1. If the hospital decides to develop MEMR in the future, it will become one of my favorite assistance tools for my work. | **□** | **□** | **□** | **□** | **□** |
| 1. Using MEMR will speed up my work (e.g. going on rounds and consulting medical records). | **□** | **□** | **□** | **□** | **□** |
| 1. Using MEMR will improve my work quality (such as enhancing the immediacy of prescribing physician orders). | **□** | **□** | **□** | **□** | **□** |
| 1. Using MEMR will make it easier to conduct my work. | **□** | **□** | **□** | **□** | **□** |
| 1. Using MEMR will improve my working performance. | **□** | **□** | **□** | **□** | **□** |
| 1. Using MEMR will help me to control my work better. | **□** | **□** | **□** | **□** | **□** |
| 1. It is easy to understand the operations of MEMR. | **□** | **□** | **□** | **□** | **□** |
| 1. It is easy to use MEMR to finish my work. | **□** | **□** | **□** | **□** | **□** |
| 1. On the whole, MEME is easy to use. | **□** | **□** | **□** | **□** | **□** |
| 1. Using MEMR may decrease my control over clinical decisions. | **□** | **□** | **□** | **□** | **□** |
| 1. Using MEMR may decrease my professional discretion over patient care decisions. | **□** | **□** | **□** | **□** | **□** |
| 1. Using MEMR can decrease my control over each step of the patient care process. | **□** | **□** | **□** | **□** | **□** |
| 1. Using MEMR may increase the monitoring of my diagnostic and therapeutic decisions by non-providers. | **□** | **□** | **□** | **□** | **□** |
| 1. Using MEMR may decrease my control over the allocation of scarce resources. | **□** | **□** | **□** | **□** | **□** |
| 1. I would find MEMR advantageous for the medical profession as a whole. | **□** | **□** | **□** | **□** | **□** |
| 1. I can access the MEMR at any time for the necessary information or service for my patient care | **□** | **□** | **□** | **□** | **□** |
| 1. I can access the MEMR anywhere for the necessary information or service for my patient care | **□** | **□** | **□** | **□** | **□** |
| 1. I can use the MEMR “anywhere,” and “anytime” at the point of patient care. | **□** | **□** | **□** | **□** | **□** |

**■ Do you have further suggestions or comments about this study?**

**________________________________________________________________________________**
